# Does Experimental Reduction of Blacklegged Tick (*Ixodes scapularis*) Abundance Reduce Lyme Disease Incidence?

**DOI:** 10.3390/pathogens12050714

**Published:** 2023-05-13

**Authors:** Richard S. Ostfeld, Felicia Keesing

**Affiliations:** 1 Cary Institute of Ecosystem Studies, Millbrook, NY 12545, USA; 2 Department of Biology, Bard College, Annandale-on-Hudson, NY 12504, USA; keesing@bard.edu

**Keywords:** acaricide, insecticide, tick control, tick management, vector control, vector management

## Abstract

Controlling the abundance of blacklegged ticks is considered the foundation for the prevention of human exposure to pathogens transmitted by these vectors in eastern North America. The use of broadcast or host-targeted acaricides is generally found to be effective at reducing the local abundance of ticks. However, studies that incorporate randomization, placebo controls, and masking, i.e., “blinding”, generally find lower efficacy. The few studies that include measurements of human–tick encounters and cases of tickborne disease have not shown impacts of acaricidal treatments. We compile literature on relevant studies from northeastern North America to address possible causes for discrepancies in study outcomes and suggest possible mechanisms that could underlie the diminished efficacy of tick control in reducing cases of tickborne disease in people.

## 1. Introduction

Humans are exposed to the causative agent of Lyme disease, *Borrelia burgdorferi*, when they are bitten by an infected ixodid tick. Thus, the presence of ixodid ticks infected with *B. burgdorferi* in habitats used by people is necessary for Lyme disease to persist in human populations. Nevertheless, within the geographic range of infected ixodid ticks, enormous variation exists in incidence rates of Lyme disease in human populations [1,2]. Understanding the causes of this variation is important for managing risk of exposure and protecting human health.

Variable risk of human exposure to Lyme disease is the result of both human behavior and tick ecology. Human behavioral factors, such as (1) the amount of time spent in tick habitat; (2) the types of activities within tick habitat; and (3) the use of personal protection while in tick habitat, e.g., protective clothing, repellents, and self-inspection, correlate with the probability of exposure to tick-borne disease [3,4]. These observations have led to public education programs aimed at reducing risk through modifying behavior [5]. Controlling the abundance of ticks is also considered an essential component of public health programs aimed at reducing the incidence of tick-borne disease [6,7,8]. The basic underlying assumption is that the probability that a person will be exposed to a tick-borne infection correlates with the abundance of ticks in the environments used by that person. If that assumption is correct, reducing the abundance of ticks should reduce human incidence of tick-borne disease regardless of variation in human behavioral factors [9].

Reductions in tick abundance can be accomplished either indirectly or directly. Indirect methods include managing habitat, e.g., vegetation and physical structure, and vertebrate hosts for ticks [1,10]. Despite plentiful public-facing advice on these indirect methods (https://www.cdc.gov/lyme/prev/in_the_yard.html, https://portal.ct.gov/-/media/caes/documents/publications/bulletins/b1010pdf) (both accessed on 7 April 2023), their efficacy is rarely quantified [4]. In contrast, the efficacy of tick control via direct methods has been well quantified. The impact of chemical and biological acaricides in reducing tick abundance has been assessed in many locations within the endemic zone of Lyme disease in North America [8,9]. Chemical acaricides that are commonly used to reduce tick abundance include synthetic pyrethroids and organophosphates, which tend to result in strong reported reductions in tick density, compared to reference sites [6]. Of the biological acaricides, entomopathogenic fungi, such as species of *Metarhizium* and *Beauveria*, are often reported to reduce tick abundance in both the lab and field [9].

Despite considerable scientific study of the effects of acaricidal treatments on tick abundance, the consequences for human exposure to tick-borne illness are rarely assessed [6,7,8,11]. Instead, it is frequently assumed, without direct evidence, that tick control will lead to a reduced incidence of tick-borne disease. Although this assumption has repeatedly been questioned [6,7,11,12,13,14,15], it still pervades the scientific literature, e.g., [16,17,18], as well as health agency recommendations to the public, e.g., https://web.uri.edu/tickencounter/, (https://www.cdc.gov/lyme/prev/in_the_yard.html, https://portal.ct.gov/-/media/caes/documents/publications/bulletins/b1010pdf, https://www.ridgefieldct.org/blast-lyme-tick-borne-disease-prevention-program, accessed on 5 May 2023). It is also reflected in the results of a recent survey of commercial tick-control operations in New York, New Jersey, and Connecticut (USA), which revealed widespread willingness of homeowners to pay roughly USD 450 per acaricide application per hectare [19].

In this paper, we review the recent scientific literature on the effects of direct tick control on human risk and incidence of tick-borne disease within the endemic zone of Lyme disease in North America. In most of North America, the competent vector is the blacklegged tick, *Ixodes scapularis*. We limit our review to experimental studies in which study areas treated to reduce blacklegged tick abundance were compared with control areas. Our primary goal is to describe the strength of evidence that experimental reductions in blacklegged tick abundance reduce human exposure to tick-borne disease.

## 2. Experimental Tick Reduction and Epidemiological Outcomes

### 2.1. Tick Reduction

The efficacy of area-wide chemical acaricides in reducing the abundance of host-seeking nymphal blacklegged ticks has repeatedly been tested in small-scale studies within residential areas. The selection of residential areas for these studies arises from the expectation that exposure to tick-borne pathogens is largely peridomestic [3,20,21]. A single application of acaricide in spring, coincident with the activity period of nymphal blacklegged ticks, is typically reported to reduce nymphal abundance by 50–100% compared either to pre-treatment abundance or to reference sites with no acaricide (e.g., [5,22,23,24,25]). Similarly, the deployment of bait boxes, devices that attract small mammals and treat them with tick-killing fipronil, have been reported to reduce the abundance of questing nymphal ticks (following a lag) by roughly 60–95% compared to field plots without bait boxes [26,27,28,29]. In addition, devices called “4-posters”, which attract white-tailed deer to a feeding station at which the deer self-apply acaricide (Amitraz), have been tested in five northeastern states and found by meta-analysis to reduce the density of host-seeking nymphal *I. scapularis* by 71% compared to sites without the devices [30]. These relatively high levels of success in controlling tick populations have suggested the promise of either area-wide or host-targeted acaricidal treatments for reducing human exposure to tick-borne diseases. Indeed, the assumption of high efficacy has contributed to public confidence that commercial pesticide applications will reliably result in a health benefit for those who occupy the treated properties (e.g., [19]).

However, the applicability of these studies to human health protection has been constrained by aspects of study design. Most studies of the ability of acaricides to control populations of host-seeking ticks compare sites treated with acaricides to reference sites with no acaricide. The number of replicate sites is often less than 10 (Table 1). Most studies do not include placebo controls. Instead, sites without acaricidal treatments are considered the “controls”. Without placebo controls, the potential impact of the act of spraying, or of deployment of bait boxes or 4-posters (with associated disturbances, including food supplementation), separate from the effect of the acaricide itself, cannot be quantified. Sometimes, the pre-treatment state within the treated sites is considered the control, such that only before–after comparisons can be made (Table 1). When control sites are used, the study design typically does not include a randomization procedure for the determination of which sites are treated and which are not. Without randomized assignment of treatment vs. control sites, subtle implicit biases about treatment assignments could lead to biased and unreliable results. This challenge is further exacerbated when “control” sites are reference areas that cannot be manipulated, e.g., forest preserves, and thus cannot be assigned a treatment. When placebo controls, e.g., the spraying of water devoid of acaricide, are used, designs typically do not incorporate experimental masking, i.e., “blinding”, meaning that this safeguard against observer bias is absent. Without masking, possible implicit biases by those collecting or reporting data cannot be assessed and eliminated, e.g., [31,32]. We recognize that other design elements differ between studies and that it is difficult to assess the importance of these differences in affecting efficacy.

To our knowledge, the first study of impacts of acaricidal treatments on tick abundance using a randomized, double-masked, placebo-controlled design was by Hinckley et al. [6] using the synthetic pyrethroid, bifenthrin. This study was unusually large, consisting of a sample of 1362 treated properties and 1365 control properties. Hinckley et al. [6] observed a 63% reduction in abundance of questing nymphal blacklegged ticks associated with the treatments, an effect that was modest compared to prior studies deploying similar synthetic pyrethroids [33,34,35]. In a separate study, testing the impact of TCS bait boxes on abundance of questing blacklegged ticks, and again using a randomized, double-masked, placebo-controlled design, Hinckley et al. [36] found no significant impact on tick abundance. This result, based on a sample of 51 experimental and 49 control properties, contrasts starkly with the strong reductions found in prior studies of bait boxes using different designs and smaller sample sizes, e.g., [26,28,29,34,37,38] (Table 1). Employing a design with randomization, placebo controls, and double-masking, Keesing et al. [39] also reported a more modest effect of bait boxes than what had been observed in many prior studies. In this case, bait boxes were associated with a ~50% reduction in the abundance of questing nymphal blacklegged ticks, with a sample of 24 residential neighborhoods (with multiple properties per neighborhood).

**Figure 1:**
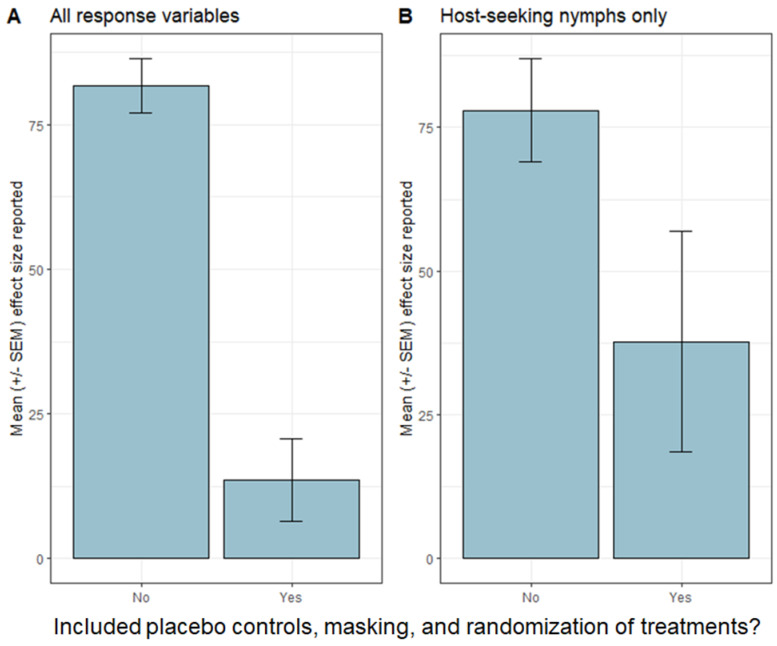
Mean effect sizes (+/- standard error of the mean), as reported in the studies listed in Table 1. Note that heterogeneity exists between studies in the ways that effect sizes are reported, and limitations in reporting of data prevent standardization of effect sizes. (**A**) shows means of all response variables given in Table 1, whereas (**B**) shows data only for the abundance of host-seeking nymphal *Ixodes scapularis* ticks.

Because design elements, such as sample size, randomization, placebo controls, and masking, are intercorrelated in studies of tick control, it is currently not possible to quantify the impacts of individual aspects of study design on the strength of effects. However, analysis of the studies in Table 1 suggests that the inclusion of randomization, placebo controls, and masking generally results in lower average effect sizes for acaricidal treatments (Figure 1). We calculated the mean reported effect size for all response variables (including both entomological and epidemiological metrics; Figure 1A) and for just the abundance of host-seeking nymphal ticks (Figure 1B). In both cases, we found stronger mean effect sizes when randomization, placebo controls, and masking were absent (Table 1). One caveat is that heterogeneity exists in how the studies report effect sizes, the consequences of which are not possible to assess given the ways data are reported. Nevertheless, the available evidence suggests that acaricidal impacts on tick abundance are systematically weaker in studies that include various safeguards against biases.

**Table 1:** Representative studies of the effects of acaricidal treatments targeted against *Ixodes scapularis* ticks in the Lyme-disease endemic zone of northeastern North America. Citations in the “Reference” column are provided in the Literature Cited section. “Location” refers to the state in the United States or province in Canada where the study took place. “Acaricidal product” refers to the product, and where relevant, the device used. In the “Placebo control?” column, “N” means that a placebo control was not used, and “Y” means a placebo control was used. The same use of “N” and “Y” applies to the use of a “Non-placebo control”, which is the use of a reference location without placebo as a “control”. The same use of “N” and “Y” applies to whether masking (=blinding) was used to prevent researchers and study subjects (when relevant) from knowing which treatments were applied to which locations, as well as to whether experimental and control locations were randomized. “Sample size” provides, the number of independent study plots/units for experimental and control treatments.

Reference	Location	Acaricidal Product	Efficacy	Placebo Control?	Non-placebo Control?	Masking (=Blinding)?	Randomization?	Sample Size (expt:cntl)
[23]	CT	carbaryl	90–100% reduction in host-seeking nymphal *I. scapularis*	N	N	N	N	5:0
[23]	CT	permethrin (“Damminix”)	31–91% reduction in immature *I. scapularis* on mice, no effect on host-seeking nymphs	N	Y	N	N	5:5
[22]	NY	carbaryl	68–78% reduction in host-seeking nymphal *I. scapularis*	N	Y	N	N	~30:24 *
[22]	NY	chlorpyrifos	87–97% reduction in host-seeking nymphal *I. scapularis*	N	Y	N	N	~30:24 *
[22]	NY	cyfluthrin	92% reduction in host-seeking nymphal *I. scapularis*	N	Y	N	N	~30:24 *
[40]	NY	permethrin (“Damminix”)	<65% reduction in immature *I. scapularis* on mice, no effect on host-seeking nymphs	N	Y	N	N	3:3
[41]	MA	permethrin (“Damminix”)	~100% reduction in immature *I. scapularis* on mice, ~100% reduction in host-seeking nymphs	N	Y	N	N	1:2
[26]	CT	fipronil (bait box)	68-84% reduction in immature *I. scapularis* on mice, 77% reduction in host-seeking adults, 64–67% reduction in infection prevalence in nymphs	N	Y	N	N	154:6
[34]	NJ	Amitraz (4-poster), deltamethrin, fipronil (bait box)	>80% reduction in immature *I. scapularis* on mice, 56–94% reduction in host-seeking nymphs	N	Y	N	N	1:1 (4-poster) 13:1 (deltametrhin, bait box)
[35]	NJ	deltamethrin	80-100% reduction in host-seeking nymphs and adults	N	Y	N	N	2:2
[30]	CT, MD, NJ, NY	Amitraz (4-poster)	71% reduction in host-seeking nymphs after 6 years (meta-analysis)	N	Y	N	N	1:1 (each state) 4:4 (altogether)
[42]	ME	bifenthrin	100% reduction in host-seeking larvae, nymphs, adults	N	Y	N	N	3:3
[33]	ME	“Eco-exempt IC2” botanical extract, bifenthin	100% reduction in host-seeking larvae, nymphs, adults	N	Y	N	N	3:3
[6]	CT, MD, NY	bifenthrin	63% reduction in host-seeking nymphs, no effect on tick encounters or cases of tick-borne disease	Y	N	Y	Y	1362:1365
[28]	NJ	fipronil (bait box)	90–97% reduction in host-seeking nymphal *I. scapularis*	N	Y	N	N	12:1
[38]	CT	fungal biocide “Met52” plus fipronil (bait box)	17-fold reduction in infected immature *I. scapularis* on mice	N	Y	N	N	6:6 (2013) 12:13 (2014–2015)
[29]	CT	fungal biocide “Met52” plus fipronil (bait box)	53% reduction in encounters with host-seeking *I. scapularis* nymphs, 77–97% reduction in host-seeking *I. scapularis*	N	Y	N	N	6:6 (2013) 12:13 (2014–2015)
[37]	CT	fungal biocide “Met52” plus fipronil (bait box)	93% reduction in probability of encountering infected *I. scapularis* nymph, 52% reduction in host-seeking nymphs	N	Y	N	N	13:12
[36]	CT	fipronil (bait box)	no effect, host-seeking nymphs, no effect infection prevalence, no effect on tick encounters or cases of tick-borne disease	Y	N	Y	Y	51:49
[43]	QU	fluralaner	68–74% reduction in immature *I. scapularis* on mice	N	Y	N	N	7:4
[39]	NY	fipronil (bait box)	~50% reduction in host-seeking *I. scapularis* nymphs, no effect on tick encounters or cases of tick-borne disease	Y	N	Y	Y	12:12
[39]	NY	fungal biocide “Met52”	No effect on host-seeking *I. scapularis* nymphs, no effect on tick encounters or cases of tick-borne disease	Y	N	Y	Y	12:12
[44]	NY	fipronil (bait box) and fungal biocide “Met52”	No effect on infection prevalence of *I. scapularis* with tick-borne pathogens	Y	N	Y	Y	12:12

* Sample size not given precisely due to unspecified eliminations of properties from the study. 24 untreated properties were used as controls for all treatments.

### 2.2. Effects on Disease Incidence

To the best of our knowledge, the study by Hinckley et al. [6] was the first to test whether experimental reduction of tick abundance resulted in a reduced rate of human encounters with ticks or of tick-borne disease in people. Hinckley et al. [6] found that a 63% reduction in the abundance of host-seeking nymphal *I. scapularis* after bifenthrin treatment was not associated with any reduction in either encounters with ticks or cases of tick-borne disease in residents of the treated properties. A later study by Keesing et al. [39] found that treatments of residential neighborhoods with TCS bait boxes, with or without the addition of the fungal biocide Met52, did not reduce encounters with ticks or the incidence of tick-borne disease in the human residents of those neighborhoods, compared to neighborhoods with placebo controls, though they did detect a significant reduction in cases of tick-borne disease in outdoor pets. Thus, the halving of nymphal tick abundance did not affect exposure of people to tick-borne disease. The use of TCS bait boxes in the study by Hinckley et al. [36] failed to reduce abundance of host-seeking nymphal *I. scapularis* and failed to affect incidence of tick-borne disease in associated human populations. We emphasize that all three of these studies employed a randomization of treatment sites, placebo controls, and masking of researchers and human subjects to treatment categories. Connally et al. [45] evaluated whether the efficacy of 4-posters in reducing human–tick encounters, and the incidence of tick-borne disease, could be rigorously tested in residential areas of NY and CT. Accounting for the large study areas required to test these devices and the human population sizes necessary to achieve sufficient statistical power, they concluded that such studies were infeasible.

Our understanding of why the observed reductions in tick populations failed to alter cases of tick-borne disease is limited. It is possible that the relationship between tick abundance and risk of human exposure to tick-borne disease is strongly nonlinear, such that reductions much greater than ~60% are required for an epidemiological effect. However, it is not clear what mechanisms might underlie such non-linearities [39]. It is possible that, even in large-scale studies with high levels of replication, the statistical power to detect an effect on disease incidence is insufficient. However, power analyses prior to the commencement of the studies by Hinckley et al. [6] and Keesing et al. [39] suggested that statistical power was adequate. Moreover, the study by Keesing et al. [39] did detect a significant reduction of bait box treatment on cases of tick-borne disease in outdoor pets, despite a lower overall number of cases in pets as compared to people. This, too, suggests adequate statistical power to detect effects when they exist. Tick-control treatments in residential areas might fail to reduce cases of tick-borne disease because patients tend to encounter the ticks that infected them outside these residential areas, e.g., in recreational areas outside their neighborhoods.

One other potential factor disconnecting direct tick control to disease incidence is human behavior. It is possible that study populations tend to use such aggressive self-protection measures, such as the use of repellents, protective clothing, and tick-checks, that reducing the size of the tick population has little added protective value. More research should be devoted to exploring these possibilities. Until the causes of the lack of efficacy of tick-control in preventing cases of tick-borne disease are better understood, it seems prudent to assume that residential tick control by the widely available commercial products may not protect residents against exposure to tick-borne infections. If residents whose properties are treated with acaricides relax their vigilance against tick bites, then the failure of tick-control interventions could have the perverse outcome of increasing exposure risk.

## 3. Discussion

The number of human encounters with ticks can reasonably be expected to correlate with the abundance of ticks in the environments used by those people. This expectation has motivated efforts to control the abundance of ticks. Several chemical and biological acaricides have been tested, generally in small-scale field trials, with levels of tick reduction suggesting considerable promise. However, when some of these same acaricides are tested in larger-scale trials, or in trials using a more rigorous study design that include randomization, placebo controls, and masking, their efficacy is generally lower and sometimes undetectable. These same larger-scale studies tend to be the only trials that quantify the impacts of tick-control on human encounters with ticks and human cases of tick-borne disease. To date, in these larger studies with measures of human impacts, no significant effects have been found. The reasons for the reduced entomological efficacy and lack of epidemiological efficacy are not clear and require active investigation. If the strong effect sizes observed arise in part from the lack of elements of study design that can minimize or eliminate implicit biases, study designs and interpretations of results should be reconsidered. We emphasize that the heterogeneity between studies in various design elements, as well as in how efficacy is estimated, complicates direct comparisons of the importance of specific aspects of study design, such as placebo controls and masking (blinding). The inclusion of randomization, placebo controls, and masking, as well as human subjects, raises the cost of research efforts to levels that are infeasible for many research groups.

We support the continued development and assessment of acaricidal compounds and devices that show promise in reducing tick populations while safeguarding the health of people, pets, wildlife, including non-target organisms, and the environment. However, we urge the designers of these studies to consider using the most rigorous study designs possible, as well as to pursue epidemiological outcomes. Funding levels for such research may need to be increased dramatically to provide the needed experimental rigor. If controlling tick abundance does not lead to associated health protections, it might not be considered worth the monetary and environmental costs. These efforts also have the potential to be counter-productive if they provide people with a false sense of safety.

As further research into acaricidal treatments proceeds, we recommend simultaneous efforts that accelerate other means of preventing tick-borne disease. These include personal-product acaricides, i.e., tick-killing soap and skin-care products, habitat management of elements, such as brush piles and invasive shrubs, in which ticks may concentrate, and vaccines against tick-borne pathogens and ticks themselves.

Despite decades of research, we still do not know where patients with tick-borne disease encountered the tick that transmitted infection. This question can be asked at several nested scales, from the habitat elements within an individual property to those in a residential neighborhood, to the local and regional hotspots for ticks that occur outside these neighborhoods. In a study of two affluent, largely white communities in CT, USA, Mead et al. [3] found that, for the 91 (of N = 934 total) participants who found a tick on them, 50% of their (self-reported) outdoor time was spent in their own or a neighbor’s yard and 50% was spent elsewhere. Time spent in one’s own or in a neighbor’s yard was associated with a ~10%–20% increase in the probability of an individual finding a tick on them, suggesting higher peridomestic risk. In contrast, individuals who reported finding ticks on multiple days within a week did not spend significantly more time in their yards than did those who found ticks on only a single day [3], suggesting the importance of some unmeasured risk factors other than peridomestic activity.

More complete knowledge of exposure locations is crucial for designing more effective acaricidal interventions. Objective (rather than self-reported) and geo-referenced determinations of human space use, in real time, combined with frequent assessments of tick encounters, may be necessary to better understand exposure locations. Such knowledge could then be used to design rigorous, site-specific tests of the efficacy of acaricidal treatments to reduce tick encounters and incidence of tick-borne disease at the most important spatial scale. In addition, determining whether the relationship between tick abundance and the probability of human exposure to tick-borne pathogens is non-linear, and if so, the density range of any inflection points, is an important research frontier. Such research would ideally explore ecological and behavioral causes for such non-linearities. The separate and combined effects of interventions aimed at tick abundance in the environment and those directed at probabilities of encountering and detecting tick contacts should be pursued. Determining whether controlling the abundance of ticks in the environment can protect human health will require an investment of resources that permits these questions to be addressed rigorously and with the highest standards of study design.

## Data Availability

New data were not collected for, and are not presented in, this contribution.
